# Supplementary material for: Postnatal Development of the Circadian Rhythmicity of Human Pineal Melatonin Synthesis and Secretion (Systematic Review)
Source: Children (Basel). 2024 Sep 29;11(10):1197. doi: 10.3390/children11101197 (PMC11506472; doi:10.3390/children11101197)
Supplement: Supplementary file 1 [file children-11-01197-s001.zip › children-3206873-supplementary-Table S1.pdf]

PRISMA 2020 Checklist

Section and Topic	Item #	Checklist item	Location where item is reported
**TITLE**			
Title	1	Identify the report as a systematic review.	The title of the paper is: “Postnatal Development of the Circadian Rhythmicity of Human Pineal Melatonin Synthesis and Secretion (Systematic Review)”
**ABSTRACT**			
Abstract	2	See the PRISMA 2020 for Abstracts checklist.	It´s oaky, see Abstract in the attached paper with Introduction, Method, Results, and Discussion.
**INTRODUCTION**			
Rationale	3	Describe the rationale for the review in the context of existing knowledge.	The present study focuses on the reasons for the physiological melatonin deficiency in the first months of life in infants in connection with the postnatal development of the pineal gland. On this pathophysiological basis, the clinical need for chrononutrition for infants is derived and justified.
Objectives	4	Provide an explicit statement of the objective(s) or question(s) the review addresses.	This paper presents the current biochemical and pathophysiological knowledge on the postnatal development of pineal melatonin synthesis, the associated signal transduction chain from the retina via the suprachiasmatic nucleus (SCN) as the master clock for diurnal timing functions, via the cervical ganglion to the provision of noradrenaline in the area of the pineal gland for the activation of pineal melatonin synthesis.
**METHODS**			
Eligibility criteria	5	Specify the inclusion and exclusion criteria for the review and how studies were grouped for the syntheses.	The questions to be examined in relation to this topic are based on clinical studies and reviews of clinical studies in humans. Data from animal experiments are used in the discussion if this allows important facts to be explored in greater depth. The limitations of transferring data from animal experiments to humans are explicitly pointed out. All languages are included in the search. Additional sources: the author has published current reviews on relevant topics [18,22,57,78–80]
Information sources	6	Specify all databases, registers, websites, organisations, reference lists and other sources searched or consulted to identify studies. Specify the date when each source was last searched or consulted.	The data from the search in PubMed on 22 August 2024 were recorded via PubMed and sent to the author by email. The author saved the lists with the search results
Search strategy	7	Present the full search strategies for all databases, registers and websites, including any filters and limits used.	On 22 August 2024, the following search strategy in PubMed returned 115 publications: ((melatonin[Title/Abstract]) AND (infant[Title/Abstract])). Only 45 articles were found under “melatonin” AND “infancy”, so the first 115 articles were examined. After excluding animal studies (16), protocols without their own data on results (2), and a retraction paper that had been reported twice (2), 95 publications remained. Of these 95 studies, following 55 studies were excluded (see Suppl. 1).
Selection process	8	Specify the methods used to decide whether a study met the inclusion criteria of the review, including how many reviewers screened each record and each report retrieved, whether they worked independently, and if applicable, details of automation tools used in the process.	Inclusion criteria: see point 6 and 7 of this PRISMA table.<br>Reviewer: N= 1 (Paditz, Ekkehart), transparency see Suppl. 1.
Data collection process	9	Specify the methods used to collect data from reports, including how many reviewers collected data from each report, whether they worked independently, any processes for obtaining or confirming data from study investigators, and if applicable, details of automation tools used in the process.	See point 6, 7, 8 o this PRISMA table and Suppl. 1 of the paper.
Data items	10a	List and define all outcomes for which data were	See table 1 of the paper.

Section and Topic	Item #	Checklist item	Location where item is reported
		sought. Specify whether all results that were compatible with each outcome domain in each study were sought (e.g. for all measures, time points, analyses), and if not, the methods used to decide which results to collect.	
	10b	List and define all other variables for which data were sought (e.g. participant and intervention characteristics, funding sources). Describe any assumptions made about any missing or unclear information.	See table 1 of the paper.
Study risk of bias assessment	11	Specify the methods used to assess risk of bias in the included studies, including details of the tool(s) used, how many reviewers assessed each study and whether they worked independently, and if applicable, details of automation tools used in the process.	See table 1 of the paper.<br>Studies in which only hypotheses were presented, rather than data, were excluded. Critical reviews on specific questions are mentioned explicitly, so that methodologically questionable studies are not inappropriately overrated.<br>The narrative review of melatonin metabolism, which was prepared with the clinical pharmacologist Prof. Dr. med. Bertolt Renner, Institut of Pharmacology and Toxicology, Medical Faculty of the Technical University in Dresden/Germany and the occupational health physician Dr. med. Marcus Bauer, Inselspital University Bern/Switzerland before, contains 51 literature references [57]. The current questions about the toxicity of melatonin in infancy have been discussed in depth with this experienced clinical pharmacologist, see Paditz et al., 2023 [57].
Effect measures	12	Specify for each outcome the effect measure(s) (e.g. risk ratio, mean difference) used in the synthesis or presentation of results.	See table 1 of the paper: point to point for each included study.
Synthesis methods	13a	Describe the processes used to decide which studies were eligible for each synthesis (e.g. tabulating the study intervention characteristics and comparing against the planned groups for each synthesis (item #5)).	See Figure 1 of the paper: Graphical aggregation and table of values of the individual measured values for melatonin in colostrum are taken from the studies by Qin (1), Aparici-Gonzalo (2), Pontes 2007 (3), Pontes 2006 (4), Honori-Franca (5), Silva (6) and Illnerova (7) as well as for mature milk from Kimata (1), Aparici-Gonzalo (2), Cohen Engler (3), Molad (4) and Silva (5) [20,37–39,94,101–104,131,132].
	13b	Describe any methods required to prepare the data for presentation or synthesis, such as handling of missing summary statistics, or data conversions.	See table 1 of the paper and Discussion with critical synthesis of the values in table 1.
	13c	Describe any methods used to tabulate or visually display results of individual studies and syntheses.	See figure 1.
	13d	Describe any methods used to synthesize results and provide a rationale for the choice(s). If meta-analysis was performed, describe the model(s), method(s) to identify the presence and extent of statistical heterogeneity, and software package(s) used.	With regard to the outcome variables, the search focuses on all available data that can contribute to answering the questions initially posed. Since anatomical, biochemical, developmental, chronobiological and sleep-related parameters are to be recorded and synthesised, this is a transdisciplinary approach. (14) Due to the heterogeneous evidence level of the available studies, the data are documented suitably and commented case-related. The results are categorised in Table 1 according to subject group, so that the risk of bias is reduced if comparable data from different author groups are available for the same topic.
	13e	Describe any methods used to explore possible causes of heterogeneity among study results (e.g. subgroup analysis, meta-regression).	See 13 d.
	13f	Describe any sensitivity analyses conducted to assess robustness of the synthesized results.	These conclusions of the paper are supported by the data presented in table 1, see

Section and Topic	Item #	Checklist item	Location where item is reported
			- the comments by Aparicio et al., 2007 [65], which have been included in table 1 in the chrononutrition category as a result of a prospective randomised study (= demand for day and night milk for infants and introduction of the term ‘chrononutrition´ for this age group),<br>- the results of the prospective cohort study by Ferber et al., 2011 [115], which showed that should not only concern the melatonin concentrations in day and night milk, but that these maturation processes correlate with noradrenergic effects on pineal mela-tonin synthesis and neuropsychological developmental parameters up to the ninth month of life (Table 1, subject group on nocturnal melatonin synthesis),<br>- the results of the longitudinal study by McMillen et al., 1993 [112], which showed a correlation between the circadian rhythms and the fact that there is a ‘greater physio-logical disruption’ of these circadian rhythms in premature infants, as well as<br>- the results of the intervention studies with control group by Cubero et al., 2006/2007 [40,41], which was reported in the introduction of the present systematic review, showed that in infants aged 4 to 20 week of life, sleep could be improved by supple-mentation with tryptophan as a precursor for melatonin formation [40,41]. The dis-advantages of artificial tryptophan supplements have already been mentioned in the introduction. This does not change the orientation towards the evolutionary established chrononutrition with human breast milk as the gold standard, which should serve as basis for breastfeeding mothers, human milk banks and infant formula manufacturers.<br><br>The present systematic review is based on several studies with a high level of evidence, such as Carballo et al., 1996, Kennaway et al., 1996, Aparicio et al., 2007, McMillen 1993, Ferber et al., 2011, Sun et al. 2024 and Sivan 2000 (Table 1), and on several reviews whose authors have made plausible fundamental conclusions (Kennaway 2000, Anderson 2017, see Table 1). Overall, the current state of knowledge regarding the necessity of using melatonin-poor day milk and melatonin-rich night milk in sense of a chrononutrition adapted to the circadian rhythm for infants is based on a compact amount of data. **In my opinion, the different quality of the studies available so far is widely compensated by the consistent tenor of majority of the studies.**
Reporting bias assessment	14	Describe any methods used to assess risk of bias due to missing results in a synthesis (arising from reporting biases).	Only those studies and reviews were included that contained specific measured values in humans. Toxicological questions that went beyond paediatric-neonatal know-how were discussed with a toxicologist and an occupational physician with experience in toxicology, so that their expertise was included in the assessment of previously unresolved toxicological questions. Critical reviews, such as Kennaway's, were explicitly cited and presented so that the debate on melatonin and sudden infant death syndrome could be evaluated on the basis of current knowledge.
Certainty assessment	15	Describe any methods used to assess certainty (or confidence) in the body of evidence for an outcome.	See 13f and 14.
**RESULTS**			
Study selection	16a	Describe the results of the search and selection process, from the number of records identified in the search to the number of studies included in the review, ideally using a flow diagram.	See flow chart and Suppl. 1 of the paper.
	16b	Cite studies that might appear to meet the inclusion criteria, but which were excluded, and explain why they were excluded.	A study with retrospective evaluation of defined geomagnetic activity patterns in temporal correlation with SIDS cases was not considered [83] (1), since this thesis by the same group of authors could not be correlated with structural changes in the pineal gland in animal experiments [84].

Section and Topic	Item #	Checklist item	Location where item is reported
			The review by Garcia-Patterson et al. 1996 [86] is not presented in Table 1, as the studies cited there by Attanasio et al. 1985 [87] and Waldhauser et al. 1988 [25] are already pre-sented in more detail in the introduction to the present paper and in Table 1.
Study characteristics	17	Cite each included study and present its characteristics.	See table 1 in detail point for point to each included study.
Risk of bias in studies	18	Present assessments of risk of bias for each included study.	See table 1 in detail point for point to each included study.
Results of individual studies	19	For all outcomes, present, for each study: (a) summary statistics for each group (where appropriate) and (b) an effect estimate and its precision (e.g. confidence/credible interval), ideally using structured tables or plots.	See table 1 in detail point for point to each included study.<br>See figure 1 of the paper.
Results of syntheses	20a	For each synthesis, briefly summarise the characteristics and risk of bias among contributing studies.	The nature of infant nutrition is, from an evolutionary biological perspective, designed for circadian-oriented 'chrononutrition' in all mammals (Figure 1). In practice, this requires that non-pooled milk be used for breastfeeding, for collecting donor milk and for the industrial production of infant formula.
	20b	Present results of all statistical syntheses conducted. If meta-analysis was done, present for each the summary estimate and its precision (e.g. confidence/credible interval) and measures of statistical heterogeneity. If comparing groups, describe the direction of the effect.	Due to the heterogeneous evidence level of the available studies, the data are documented suitably and commented case-related. The results are categorised in Table 1 according to subject group, so that the risk of bias is reduced if comparable data from different author groups are available for the same topic.
	20c	Present results of all investigations of possible causes of heterogeneity among study results.	See 20b.
	20d	Present results of all sensitivity analyses conducted to assess the robustness of the synthesized results.	See 20b.
Reporting biases	21	Present assessments of risk of bias due to missing results (arising from reporting biases) for each synthesis assessed.	Only those studies and reviews were included that contained specific measured values in humans.
Certainty of evidence	22	Present assessments of certainty (or confidence) in the body of evidence for each outcome assessed.	See 20b and table 1 point to point for each included study.
**DISCUSSION**			
Discussion	23a	Provide a general interpretation of the results in the context of other evidence.	Based on the histologically detectable developmental and differentiation steps described by Meduna [136] and Min in infants, the same as in animal studies [140–142] it can be assumed that the cause of the delayed development of the ability of the pineal gland to synthesise melatonin in the first months of life lies in the development of the pineal gland itself, which only begins postnatally. Since animal data cannot be extrapolated to humans without further investigation, further studies are needed to clarify how postnatal differentiation and connection of the pinealocytes with the associated structures of the signal transduction chain from the retina to the pineal gland occurs in humans. Lhx4, noradrenaline and timezyme are likely to continue to play a leading role in this.
	23b	Discuss any limitations of the evidence included in the review.	See 20b.
	23c	Discuss any limitations of the review processes used.	The research and development of the present publication was carried out by one author. However, his approach was presented very transparently in Suppl. 1 and in Table 1, as well as in the chapter

PRISMA 2020 Checklist

Section and Topic	Item #	Checklist item	Location where item is reported
			on methodology. Questions from borderline areas such as toxicology were discussed and published in previously published papers with an experienced toxicologist and an experienced occupational physician, see above.
	23d	Discuss implications of the results for practice, policy, and future research.	Further research is needed to examine the following open questions in more detail: Which other genetic programmes play a role in the maturation of the pineal gland's functions after birth? How can the already known and well-documented knowledge about in-creasing melatonin concentrations in cows' night milk be taken into account in practice by specifically improving the lighting during the day and at night in cattle sheds? How can the milking practices of milk producers be changed to produce melatonin-rich night milk that would allow the industrial production of chrononutrition for infants? What communication strategies are effective in educating breastfeeding mothers that milk should not be pooled? This challenge also applies to breast milk collection centres. Laughter, positive thinking, restful sleep and sensible light exposure can increase the melatonin content of breast milk, so this knowledge should be integrated into communication and support for the parents of infants. Before clinical conclusions are drawn from the open questions ad-dressed here, the critical remarks published by Boutin, Kennaway and Jockers regarding the interpretation of results in relation to melatonin [190,191] should be repeatedly re-viewed and taken into account. This standard has been applied to the present systematic review, since initially published associations between sleep disorders in infants were re-ported, the quality of these findings was then evaluated with the results of place-bo-controlled intervention studies, so that qualitative and quantitative relationships can then be assumed.
**OTHER INFORMATION**			
Registration and protocol	24a	Provide registration information for the review, including register name and registration number, or state that the review was not registered.	Registration: Non.
	24b	Indicate where the review protocol can be accessed, or state that a protocol was not prepared.	I explained that the entire review process can be disclosed; see the editorial documents of the journal 'children'.
	24c	Describe and explain any amendments to information provided at registration or in the protocol.	See Suppl. 1 of the paper.
Support	25	Describe sources of financial or non-financial support for the review, and the role of the funders or sponsors in the review.	There were no financial contributions. The idea of this systematic review comes from the author alone. The text was created by the author alone, without outside help or the use of artificial intelligence.
Competing interests	26	Declare any competing interests of review authors.	The author declares that there are no conflicts of interest. He is a voting member of the Ethics Commission of the Technical University of Dresden, managing partner of kleanthes Verlag für Medizin und Prävention GmbH & Co. KG, Dresden, and author of the patent application cited in this article [73].
Availability of data, code and other materials	27	Report which of the following are publicly available and where they can be found: template data collection forms; data extracted from included studies; data used for all analyses; analytic code; any other materials used in the review.	See 7.<br>The narrative review of melatonin metabolism, which was prepared with the clinical pharmacologist Prof. Dr. med. Bertolt Renner, Institut of Pharmacology and Toxicology, Medical Faculty of the Technical University in Dresden/Germany and the occupational health physician Dr. med. Marcus Bauer, Inselspital University Bern/Switzerland before, contains 51 literature references [57]. The current questions about the toxicity of melatonin in infancy have been discussed in depth with this experienced clinical pharmacologist, see Paditz et al., 2023 [57].

PRISMA 2020 Checklist

From: Page MJ, McKenzie JE, Bossuyt PM, Boutron I, Hoffmann TC, Mulrow CD, et al. The PRISMA 2020 statement: an updated guideline for reporting systematic reviews. BMJ 2021;372:n71. doi: 10.1136/bmj.n71
