# Supplementary material for: Postnatal Development of the Circadian Rhythmicity of Human Pineal Melatonin Synthesis and Secretion (Systematic Review)
Source: Children (Basel). 2024 Sep 29;11(10):1197. doi: 10.3390/children11101197 (PMC11506472; doi:10.3390/children11101197)
Supplement: Supplementary file 1 [file children-11-01197-s001.zip › children-3206873Supplement 1_List of excluded papers_Postnatal development of human pineal melatonin-S1.pdf]

## Suppl. 1

## List of studies extracted from the remaining 95 studies

(On 22 August 2024, the following search strategy in **PubMed returned 115 publications**: ((melatonin[Title/Abstract]) AND (infant[Title/Abstract])). Only 45 articles were found under "melatonin" AND "infancy", so the first 115 articles were examined. After excluding animal studies (16), protocols without their own data on results (2), and a retraction paper that had been reported twice (2), **95 publications remained**.

**Following studies were excluded:**

| Topic (number of studies) [source] | Explanation | | |
|---|---|---|---|
| | Different topic | Theses without measured values | N |
| Additional study protocols without data (2) [1, 2] | | Ø | 2 |
| related to children with Smith-Magenis syndrome (1)[3], | Ø | | 1 |
| sleep and breathing disorders in FBXO11 and RAF1 mutations (1)[4], | Ø | | 1 |
| 5 year old boy with tobacco embryopathy, autism spectrum disorder, ADHD and insomnia (1)[5], | Ø | | 1 |
| neonatal asphyxia or perinatally acquired brain damage (11)[6-16], | Ø | | 11 |
| general advantages of tryptphan without specific data on infants (1)[17], | Ø | Ø | 1 |
| treatment of pain (2)[18, 19], | Ø | | 2 |
| prophylaxis of bronchopulmonary dysplasia [20] or later cardiovascular complications [21] or later borderline personality disorders [22] (3), | Ø | | 3 |
| effects of maternal obesity on the composition of colostrum (2)[23, 24], | Ø | | 2 |
| in vitro studies on the activation of phagocytosis by melatonin in colostrum cells respectively on the effects of melatonin on mononuclear cells in umbilical cord blood (2)[25, 26], | Ø | | 2 |
| effects of circadian-adapted lighting in a neonatal intensive care unit (1)[27], | Ø | | 1 |
| postpartum depression or postpartum fatigue in mothers (2)[28, 29], | Ø | Ø | 2 |
| maternal magnesium deficiency (3)[30-32], | Ø | Ø | 3 |
| speculations without measured values about yin-yang models and noradrenaline with references to numerous diseases (1)[33], and about stroke and coronary heart disease (1)[34], | Ø | Ø | 2 |
| theses without a corresponding database on 'melatonin dysregulation' as a 'causal factor' for autism spectrum and ADHD [35] (1) or sudden infant death syndrome (8)[36-43], | Ø | Ø | 9 |
| study with healthy control group examining sleep architecture after acute cyanotic apnoea without data on melatonin (1)[44], | Ø | Ø | 1 |
| theses without data on the influence of calcium, serotonin and melatonin on infant colic (3)[45-47], | Ø | Ø | 3 |
| report on the diagnosis of cow's milk allergy (1)[48], | Ø | | 1 |

| | | | |
|---|---|---|---|
| reports on physiology of puberty (2)[49, 50], | Ø | | 2 |
| theses on manic states in mothers (1)[51], | Ø | Ø | 1 |
| effect of season on the sleep architecture of infants without estimation light exposure and the melatonin levels (1)[52], | Ø | Ø | 1 |
| investigation of the diurnal variation in the frequency of the time of delivery (1)[53] and | Ø | Ø | 1 |
| effect of supplementation the nutrition with tryptophan, adenosine und uridine bei Säuglingen im Alter von 8-16 Monaten [54], cited by Friedman 2018 (1) [17]. | Ø | | 1 |
| A study with retrospective evaluation of defined geomagnetic activity patterns in temporal correlation with SIDS cases was not considered (1)[55].* | Ø | | 1 |
| ∑ | | | **55** |

*) Excluded, since this thesis by the same group of authors could not be correlated with structural changes in the pineal gland in animal experiments [56].

**References**


[1] Garofoli F, Longo S, Pisoni C, Accorsi P, Angelini M, Aversa S, Caporali C, Cociglio S, De Silvestri A, Fazzi E, Rizzo V, Tzialla C, Zecca M, Orcesi S: Oral melatonin as a new tool for neuroprotection in preterm newborns: study protocol for a randomized controlled trial. Trials 2021;22:82.

[2] Kaur S, Teoh AN, Shukri NHM, Shafie SR, Bustami NA, Takahashi M, Lim PJ, Shibata S: Circadian rhythm and its association with birth and infant outcomes: research protocol of a prospective cohort study. BMC Pregnancy Childbirth 2020;20:96.

[3] Gropman AL, Duncan WC, Smith AC: Neurologic and developmental features of the Smith-Magenis syndrome (del 17p11.2). Pediatric neurology 2006;34:337-350.

[4] Gleason E, Malik K, Sannar E, Kamara D, Serrano V, Augustyn M: Challenging Case: A Multidisciplinary Approach to Demystifying Chronic Sleep Impairment in an Infant with a Complex Medical and Behavioral Profile. Journal of developmental and behavioral pediatrics : JDBP 2024;45:e176-e179.

[5] Genovese A, Moore T, Haynes PC, Augustyn M: Interoception in Practice: The Gut-Brain Connection. Journal of developmental and behavioral pediatrics : JDBP 2022;43:489-491.

[6] Malhotra A, Rocha A, Yawno T, Sutherland AE, Allison BJ, Nitsos I, Pham Y, Jenkin G, Castillo-Melendez M, Miller SL: Neuroprotective effects of maternal melatonin administration in early-onset placental insufficiency and fetal growth restriction. Pediatric research 2024;95:1510-1518.

[7] Cannavò L, Perrone S, Gitto E: Brain-Oriented Strategies for Neuroprotection of Asphyxiated Newborns in the First Hours of Life. Pediatric neurology 2023;143:44-49.

[8] Xie Y, Yang Y, Yuan T: Brain Damage in the Preterm Infant: Clinical Aspects and Recent Progress in the Prevention and Treatment. CNS & neurological disorders drug targets 2023;22:27-40.

[9] Victor S, Rocha-Ferreira E, Rahim A, Hagberg H, Edwards D: New possibilities for neuroprotection in neonatal hypoxic-ischemic encephalopathy. European journal of pediatrics 2022;181:875-887.

[10] Yates N, Gunn AJ, Bennet L, Dhillon SK, Davidson JO: Preventing Brain Injury in the Preterm Infant-Current Controversies and Potential Therapies. International journal of molecular sciences 2021;22.

[11] D'Angelo G, Chimenz R, Reiter RJ, Gitto E: Use of Melatonin in Oxidative Stress Related Neonatal Diseases. Antioxidants (Basel, Switzerland) 2020;9.

[12] Solevåg AL, Schmölzer GM, Cheung PY: Novel interventions to reduce oxidative-stress related brain injury in neonatal asphyxia. Free radical biology & medicine 2019;142:113-122.

[13] Denihan NM, Kirwan JA, Walsh BH, Dunn WB, Broadhurst DI, Boylan GB, Murray DM: Untargeted metabolomic analysis and pathway discovery in perinatal asphyxia and hypoxic-ischaemic encephalopathy. Journal of cerebral blood flow and metabolism : official journal of the International Society of Cerebral Blood Flow and Metabolism 2019;39:147-162.

[14] Barton SK, Tolcos M, Miller SL, Christoph-Roehr C, Schmölzer GM, Moss TJ, Hooper SB, Wallace EM, Polglase GR: Ventilation-Induced Brain Injury in Preterm Neonates: A Review of Potential Therapies. Neonatology 2016;110:155-162.

[15] Wilkinson D, Shepherd E, Wallace EM: Melatonin for women in pregnancy for neuroprotection of the fetus. The Cochrane database of systematic reviews 2016;3:Cd010527.

[16] Galland BC, Elder DE, Taylor BJ: Interventions with a sleep outcome for children with cerebral palsy or a post-traumatic brain injury: a systematic review. Sleep Med Rev 2012;16:561-573.

[17] Friedman M: Analysis, Nutrition, and Health Benefits of Tryptophan. International journal of tryptophan research : IJTR 2018;11:1178646918802282.

[18] Behura SS, Dhanawat A, Nayak B, Panda SK: Comparison between oral melatonin and 24% sucrose for pain management during retinopathy of prematurity screening: a randomized controlled trial. The Turkish journal of pediatrics 2022;64:1013-1020.

[19] Gitto E, Aversa S, Salpietro CD, Barberi I, Arrigo T, Trimarchi G, Reiter RJ, Pellegrino S: Pain in neonatal intensive care: role of melatonin as an analgesic antioxidant. Journal of pineal research 2012;52:291-295.

[20] Yang X, Jiang S, Deng X, Luo Z, Chen A, Yu R: Effects of Antioxidants in Human Milk on Bronchopulmonary Dysplasia Prevention and Treatment: A Review. Front Nutr 2022;9:924036.

[21] Gombert M, Codoñer-Franch P: Melatonin in Early Nutrition: Long-Term Effects on Cardiovascular System. International journal of molecular sciences 2021;22.

[22] Anderson G: Pathoetiology and pathophysiology of borderline personality: Role of prenatal factors, gut microbiome, mu- and kappa-opioid receptors in amygdala-PFC interactions. Progress in neuro-psychopharmacology & biological psychiatry 2020;98:109782.

[23] Pereira G, Morais TC, França EL, Daboin BEG, Bezerra IMP, Pessoa RS, de Quental OB, Honório-França AC, Abreu LC: Leptin, Adiponectin, and Melatonin Modulate Colostrum Lymphocytes in Mothers with Obesity. International journal of molecular sciences 2023;24.

[24] Morais TC, Honorio-França AC, Fujimori M, de Quental OB, Pessoa RS, França EL, de Abreu LC: Melatonin Action on the Activity of Phagocytes from the Colostrum of Obese Women. Medicina (Kaunas, Lithuania) 2019;55.

[25] Pires-Lapa MA, Tamura EK, Salustiano EM, Markus RP: Melatonin synthesis in human colostrum mononuclear cells enhances dectin-1-mediated phagocytosis by mononuclear cells. Journal of pineal research 2013;55:240-246.

[26] Zhou W, Wang P, Tao L: Effect of melatonin on proliferation of neonatal cord blood mononuclear cells. World journal of pediatrics : WJP 2009;5:300-303.

[27] Vásquez-Ruiz S, Maya-Barrios JA, Torres-Narváez P, Vega-Martínez BR, Rojas-Granados A, Escobar C, Angeles-Castellanos M: A light/dark cycle in the NICU accelerates body weight gain and shortens time to discharge in preterm infants. Early human development 2014;90:535-540.

[28] Anderson G, Maes M: Postpartum depression: psychoneuroimmunological underpinnings and treatment. Neuropsychiatric disease and treatment 2013;9:277-287.

[29] Groër M, Davis M, Casey K, Short B, Smith K, Groër S: Neuroendocrine and immune relationships in postpartum fatigue. MCN The American journal of maternal child nursing 2005;30:133-138.

[30] Durlach J, Pagès N, Bac P, Bara M, Guiet-Bara A: New data on the importance of gestational Mg deficiency. Magnesium research 2004;17:116-125.

[31] Durlach J: New data on the importance of gestational Mg deficiency. Journal of the American College of Nutrition 2004;23:694s-700s.

[32] Durlach J, Pagès N, Bac P, Bara M, Guiet-Bara A: Biorhythms and possible central regulation of magnesium status, phototherapy, darkness therapy and chronopathological forms of magnesium depletion. Magnesium research 2002;15:49-66.

[33] Backon J: Inhibiting noradrenergic overactivity by inhibition of thromboxane and concomitant activation of opiate receptors via dietary means. Med Hypotheses 1989;29:65-74.

[34] Maurizi CP: Short note: The fetal origins hypothesis: linking pineal gland hypoplasia with coronary heart disease and stroke. Med Hypotheses 1998;50:357-358.

[35] Hellmer K, Nyström P: Infant acetylcholine, dopamine, and melatonin dysregulation: Neonatal biomarkers and causal factors for ASD and ADHD phenotypes. Med Hypotheses 2017;100:64-66.

[36] Goldwater PN, Oberg EO: Infection, Celestial Influences, and Sudden Infant Death Syndrome: A New Paradigm. Cureus 2021;13:e17449.

[37] Durlach J, Pagès N, Bac P, Bara M, Guiet-Bara A: Importance of magnesium depletion with hypofunction of the biological clock in the pathophysiology of headhaches with photophobia, sudden infant death and some clinical forms of multiple sclerosis. Magnesium research 2004;17:314-326.

[38] Durlach J, Pagès N, Bac P, Bara M, Guiet-Bara A: Magnesium deficit and sudden infant death syndrome (SIDS): SIDS due to magnesium deficiency and SIDS due to various forms of magnesium depletion: possible importance of the chronopathological form. Magnesium research 2002;15:269-278.

[39] Paquette H: The pineal gland. Neonatal network : NN 2000;19:9-11.

[40] Maurizi CP: Could exogenous melatonin prevent sudden infant death syndrome? Med Hypotheses 1997;49:425-427.

[41] Weissbluth M: Melatonin increases cyclic guanosine monophosphate: biochemical effects mediated by porphyrins, calcium and nitric oxide. Relationships to infant colic and the Sudden Infant Death Syndrome. Med Hypotheses 1994;42:390-392.

[42] Weissbluth L, Weissbluth M: Sudden infant death syndrome: a genetically determined impaired maturation of the photoneuroendocrine system. A unifying hypothesis. Journal of theoretical biology 1994;167:13-25.

[43] Maurizi CP: Could supplementary dietary tryptophan prevent sudden infant death syndrome? Med Hypotheses 1985;17:149-154.

[44] Cornwell AC, Feigenbaum P, Kim A: SIDS, abnormal nighttime REM sleep and CNS immaturity. Neuropediatrics 1998;29:72-79.

[45] Weissbluth L, Weissbluth M: The photo-biochemical basis of infant colic: pineal intracellular calcium concentrations controlled by light, melatonin, and serotonin. Med Hypotheses 1993;40:158-164.

[46] Weissbluth L, Weissbluth M: Infant colic: the effect of serotonin and melatonin circadian rhythms on the intestinal smooth muscle. Med Hypotheses 1992;39:164-167.

[47] Weissbluth M, Weissbluth L: Colic, sleep inertia, melatonin and circannual rhythms. Med Hypotheses 1992;38:224-228.

[48] Pajno GB, Barberio F, Vita D, Caminiti L, Capristo C, Adelardi S, Zirilli G: Diagnosis of cow's milk allergy avoided melatonin intake in infant with insomnia. Sleep 2004;27:1420-1421.

[49] Sizonenko PC: Physiology of puberty. Journal of endocrinological investigation 1989;12:59-63.

[50] Sizonenko PC: Normal sexual maturation. Pediatrician 1987;14:191-201.

[51] Maurizi CP: A preliminary understanding of mania: roles for melatonin, vasotocin and rapid-eye-movement sleep. Med Hypotheses 2000;54:26-29.

[52] Kärki A, Paavonen EJ, Satomaa AL, Saarenpää-Heikkilä O, Himanen SL: Sleep architecture is related to the season of PSG recording in 8-month-old infants. Chronobiology international 2020;37:921-934.

[53] Chaney C, Goetz TG, Valeggia C: A time to be born: Variation in the hour of birth in a rural population of Northern Argentina. American journal of physical anthropology 2018;166:975-978.

[54] Cubero J, Chanclón B, Sánchez S, Rivero M, Rodríguez AB, Barriga C: Improving the quality of infant sleep through the inclusion at supper of cereals enriched with tryptophan, adenosine-5'-phosphate, and uridine-5'-phosphate. Nutritional neuroscience 2009;12:272-280.

[55] O'Connor RP, Persinger MA: Geophysical variables and behavior: LXXXII. A strong association between sudden infant death syndrome and increments of global geomagnetic activity--possible support for the melatonin hypothesis. Perceptual and motor skills 1997;84:395-402.

[56] Dupont MJ, McKay BE, Parker G, Persinger MA: Geophysical variables and behavior: XCIX. Reductions in numbers of neurons within the parasolitary nucleus in rats exposed perinatally to a magnetic pattern designed to imitate geomagnetic continuous pulsations: implications for sudden infant death. Perceptual and motor skills 2004;98:958-966.
